# On the Way to *in vitro* Platelet Production

**DOI:** 10.3389/fmed.2018.00239

**Published:** 2018-08-28

**Authors:** Catherine Strassel, Christian Gachet, François Lanza

**Affiliations:** Université de Strasbourg, INSERM, EFS Grand Est, BPPS UMR-S 1255, FMTS, Strasbourg, France

**Keywords:** platelets, *in vitro* production, megakaryocytes, biomanufacturing, hematopoietic stem cells

## Abstract

The severely decreased platelet counts (10–30. 10^3^ platelets/μL) frequently observed in patients undergoing chemotherapy, radiation treatment, or organ transplantation are associated with life-threatening increased bleeding risks. To circumvent these risks, platelet transfusion remains the treatment of choice, despite some limitations which include a limited shelf-life, storage-related deterioration, the development of alloantibodies in recipients and the transmission of infectious diseases. A sustained demand has evolved in recent years for controlled blood products, free of infectious, inflammatory, and immune risks. As a consequence, the challenge for blood centers in the near future will be to ensure an adequate supply of blood platelets, which calls for a reassessment of our transfusion models. To meet this challenge, many laboratories are now turning their research efforts toward the *in vitro* and customized production of blood platelets. In recent years, there has been a major enthusiasm for the cultured platelet production, as illustrated by the number of reviews that have appeared in recent years. The focus of the present review is to critically asses the arguments put forward in support of the culture of platelets for transfusion purposes. In light of this, we will recapitulate the main advances in this quickly evolving field, while noting the technical limitations to overcome to make cultured platelet a transfusional alternative.

## Introduction

Blood platelets are small anucleate cells (2–4 μm in diameter) derived from the cytoplasmic fragmentation of their MK precursor (1). MKs are produced in the bone marrow through a highly orchestrated process (2). Hematopoietic stem cells (HSCs) lie at the apex of this process and give rise to progenitors which progressively commit to the megakaryocytic lineage to produce immature MKs (3). MK maturation involves an increase in DNA content (up to 64N) through endomitosis accompanied by massive enlargement of the cytoplasm, the emergence of numerous alpha and dense granules and the development of an extensive membrane network, the demarcation membrane system (DMS) (4–6). Terminally differentiated MKs are intimately associated with the sinusoidal endothelium of the bone marrow. Following extensive cytoskeletal remodeling, fully mature MKs extend cytoplasmic projections called proplatelets into the vessel lumen, where platelets are released under shear forces produced by the circulating blood (7, 8). The entire sequence is strongly influenced by cytokines, extracellular matrix components, surface topography, matrix stiffness, and blood flow (9). This efficient procedure generates 10^11^ functional platelets per day to sustain an average count of 3.10^11^ platelets/L in man (10).

## The cultured platelets in the transfusional context

More than 100 million blood donations are collected each year, but the transfusion situation varies greatly in different parts of the world. Nearly half of the donations are made in high-income countries, where <20% of the world's population lives (WHO). In industrialized countries, blood banks operate on a just-in-time basis. Maintaining an adequate platelet supply, ensuring their appropriate use and guaranteeing transfusion safety, together with the prevention of the transmission of infectious diseases, are the main concerns of these blood banks.

In this context, the field of platelet and transfusion research has witnessed an increasing interest in producing platelets *in vitro*. A number of arguments are frequently put forward to justify this research on the grounds of three main threats: i) a risk of shortage, ii) the contamination hazard, and iii) the immunological risk.

- The shortage threat: Maintaining appropriate stocks of platelet concentrates is becoming a major concern worldwide, due to the ever increasing number of patients experiencing long periods of severe thrombocytopenia related to bone marrow failure, anti-cancer therapy, bone marrow grafts, or immune-related or drug-induced thrombocytopenia (11). The short *in vivo* half-life of human platelets imposes regular platelet transfusions for these patients, while a maximum shelf-life of 5 days further increases the demand for platelets. In the USA, platelet transfusion rose by 7.3% from 2008 to 2011 and the market for platelets is expected to grow at a rate of 5.3% per annum over the next decade (12). This enhanced need has been cited to advocate the development of *in vitro* platelet production, although these figures might not apply equally to all countries. In France, for example, platelet transfusion increased by only 0.5% from 2012 to 2016 and has remained stable since, principally due to new guidelines allowing a reduction in the number of transfused platelets per unit body weight (13). Whereas this has shelved the prospect of a short term shortage, the long term trend merits surveillance. In any event, all countries are facing situations with peak demands and/or periods of low blood donation (vacations, public holidays…) where cultured platelets could represent a real alternative to maintain optimal stocks of platelet concentrates.
- The contamination hazard: Platelet transfusion has been routine practice for over five decades (14) but is however not devoid of potential risks. A bacterial contamination remains the major cause of platelet transfusion-related morbidity and mortality (15). Fortunately, the introduction of pathogen inactivation systems and bacterial detection tests, together with careful donor screening and rigorous skin disinfection, has raised transfusion safety to levels never achieved before (16). Nevertheless, the risks of biological hazards and contamination of blood products cannot be totally eliminated and also vary widely between countries. Platelets can capture emergent pathogens which remain undetectable or possibly resistant to inactivation, leading to a residual risk of infection (17). To circumvent these drawbacks and reach conditions of absolute safety, cultured platelets could be an attractive alternative.
- The immunological risk: Alloimmunization and platelet refractoriness remain major complications associated with platelet transfusion, despite the introduction of leukodepletion methods (18). The selection of HLA-compatible platelets and/or crossmatch-negative donors can solve these problems but is often difficult to achieve (19). In addition to alloimmunization, ABO-incompatibility can result in weaker transfusion efficacy (20). These problems could be resolved by the generation of universal cultured platelets lacking HLA class I and expressing preferably 0 antigens to improve their compatibility (21).

In summary, although platelet transfusion remains self-sustainable and safe, transfusion practices are destined to evolve, justifying as a precautionary measure research focusing on the efficient culture of platelets. The availability of cultured platelets, free of infectious, inflammatory, and immune risks, would undoubtedly be a real step forward for patients requiring frequent blood transfusions or lacking suitable compatible donors.

## Overview of the challenge

**Figure 1:**
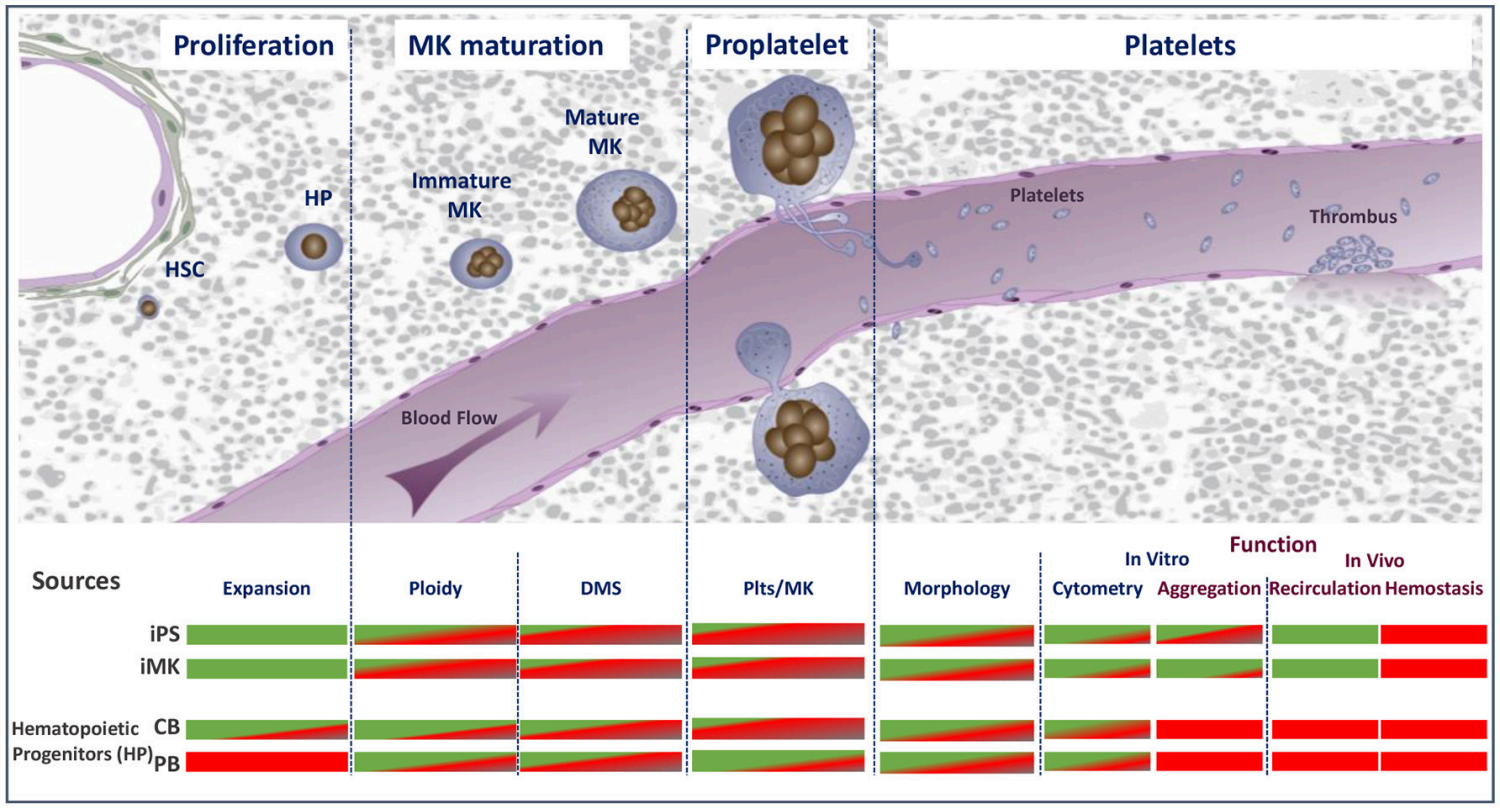
Schematic representation of the major stages of platelet biogenesis coupled with an overview of the main technical or biological hurdles that have either been overcome (green) or need to be met (red) to consider cultured platelet as a clinical alternative. HSC, hematopoietic stem cells; HP, hematopoietic progenitors; MK, megakaryocytes; DMS, demarcation membrane system; Plts, platelets; iPS, induced pluripotent stem cells; iMK, immortalized megakaryocytes.

Platelet culture for transfusion will be quite a challenging task. It will require their production in amounts equivalent to one unit (2–5.10^11^) of apheresis- or buffy coat-derived platelets and with the quality and functionality of native platelets. In essence, the *in vitro* conditions need to reproduce as closely as possible the *in vivo* environment. Assuming that each bone marrow megakaryocyte (MK) generates 2000–3000 platelets, 250.10^6^ mature MKs will be needed to produce one unit of platelet concentrate. However, despite an increasing knowledge of the molecular and cellular mechanisms governing platelet production and the development of innovative bioreactor technologies, the current yields have remained limited to 100 to 150 platelets/MK over the past several years (22, 23). To meet the challenge still ahead, there is a need to develop further knowledge (Figure 1).

1. To reach sufficient MK progenitor amplification efficiencies to obtain the equivalent of one unit of platelets (~5.10^11^ platelets);
2. To obtain a level of MK maturation closely matching that of the bone marrow;
3. To efficiently release platelets from mature MKs;
4. To demonstrate native hemostatic properties and functionality following transfusion.

## Improving MK amplification efficiencies

The source of hematopoietic progenitors/stem cells is of paramount importance and conditions the strategies and expansion capacities. Two main sources have been used i) pluripotent stem cells, including human embryonic stem cells (hESCs) and induced pluripotent stem cells (iPSCs), and ii) hematopoietic progenitors derived from bone marrow (BM), cord blood (CB) and peripheral blood (PB) (CD34+ cells). Each of these offers advantages and disadvantages for the development of a transfusion product.

- Pluripotent stem cells: hESCs and iPSCs both possess the significant advantage of a self-renewal capacity. iPSCs offer the additional benefit of avoiding the ethical concerns raised by hESCs and therefore constitute the most attractive pluripotent stem cells (24). The reader can refer to two previous excellent reviews on this subject (25, 26). Significant progress has been made in iPSC engineering to enhance platelet production. A promising development has undoubtedly been the generation of two types of expandable MK line (25). One type was obtained following several optimization steps resulting in the sequential introduction of c-MYC, BMI1, and BCL-XL (27). The second was developed by overexpressing the transcription factors GATA-1, FLI1, and TAL1 under chemically defined conditions (28). Both cell lines tolerate cryopreservation and can be expanded upon demand to generate platelets with higher efficiency and in shorter times as compared to iPSC-derived MKs. In the objective of avoiding platelet transfusion refractoriness, another remarkable achievement has been the generation of iPSC-derived HLA class I-silenced MKs and platelets using RNA-interference TALEN or *CRISPR/Cas9* editing strategies (29–31).
  Although the above arguments speak in favor of iPSCs, this source still faces a number of drawbacks. The yield of platelets remains low with < 10 platelets/MK, possibly due to some immaturity of iPSC-derived MKs (low ploidy and a less well developed DMS) (25, 27, 28, 32). Another drawback for clinical applications is the potential tumorigenicity of these cells. This risk is considered to be minor on the grounds that platelets lack replication and can be irradiated before transfusion (22, 25). However, caution might prevail and impede their acceptance by regulatory authorities. In any case, careful separation of the *bona fide* platelets from other cellular elements, such as nucleated cells including immature MK, large fragments with remnant nuclear material (DNA, RNA), in the final culture suspension will be required to minimize gene-related risks.
- Hematopoietic progenitors: As compared to hESCs and iPSCs, hematopoietic progenitors, conventionally isolated through their CD34 positivity, are technologically easier to manage for platelet generation. They can be derived from cord blood (CB), bone marrow (BM) or peripheral blood (PB) and their harvest is easy and rapid with no ethical concerns and low cost (33). The major advantage of CD34+ cells is their platelet yield, usually around 100–150 platelets/MK, while several studies have mentioned that platelets derived from these progenitors share ultrastructural and functional characteristics with circulating platelets (34, 35).

One limitation often evoked to oppose the use of CD34+ cells is their finite expansion. However, large numbers of safe CB-derived HSCs are stored around the world and could be used for the bio-manufacture of platelets. Similarly, PB-derived CD34+ cells eluted from leukoreduction filters (LRFs) represent a source with strong potential. Around 0.4.10^6^ PB-derived CD34+ cells can be eluted from one LRF and the French Blood Bank, for example, destroys more than 3.10^6^ LRFs/year (36). LRFs are an easily available and safe source of cells submitted to a stringent quality control process. Automation and standardization of the process would allow the constitution of large homogeneous and safe CD34+ cell pools. Even if these cells do not possess the theoretical unlimited expansion potential of iPSCs, recent studies indicate that this could be partly overcome by the use of novel agents like StemRegenin 1 (SR1) (37), nicotinamide (NAM) (38), mesenchymal stromal cell (MSC) coculture (39), or notch ligands (40). Moreover, CD34+ cells harbor the same potential as iPSCs to produce HLA-deficient platelets, since both CB- and PB-derived HSCs could be selected and pooled according to their HLA/ABO phenotype to generate compatible platelets for transfusion. Altogether, the availability of safe, HLA/ABO-pooled, CB-, and LRF-derived CD34+ cells combined with new strategies favoring their proliferation could lead to a regain of the use of CD34+ cells for platelet production.

Choosing between iPSCs and hematopoietic progenitors will be a matter of compromise taking into account the proliferation and maturation potentials of the cells when planning large-scale cultures. Despite undeniable technological advances, platelet production from iPSCs requires relatively complex and sophisticated methods, which might complicate the industrial-scale generation of cultured platelets. We postulate that CD34+ cells derived from PB, which are presently underexploited, could represent an interesting trade-off in terms of their availability, cost, and MK maturation and platelet yields (35).

## Obtaining a level of MK maturation closely matching that of the BM

Efficient platelet production requires a high degree of MK maturation, which is itself dependent on i) efficient endomitosis and ii) DMS expansion (2). Endomitosis contributes to the production of large amounts of proteins and to organelle development within a single MK (6). DMS expansion provides the reservoir of membranes required to feed the extension of numerous proplatelets and *in fine* the release of individual platelets. To reach an optimal degree of MK maturation *in vitro* we have to faithfully mimic these processes, which are influenced by specific microenvironments in the bone marrow (cytokines, stiffness) and depend on efficient lipid biosynthesis.

- Ploidization. So far, even under optimal conditions, MKs derived from adult progenitors or iPSCs present lower ploidy levels than MKs resident in the bone marrow, indicating a certain lack of maturity. Consistent with this finding, it has been observed that smaller and less polyploid MKs produce fewer platelets than larger MKs (41). This has also been documented in fetal and neonatal MKs, which are significantly smaller and of lower ploidy than adult MKs and produce fewer platelets than their adult counterparts (42). Whereas the mechanisms underlying the small size and low ploidy of neonatal MKs remain unclear, Elagib et al. recently identified an RNA-binding protein, IGF2BP3, regulating the human fetal-adult MK transition (43). These authors demonstrated that downregulation of IGF2BP3 using a lentiviral strategy enhanced neonatal MK enlargement, growth arrest, and polyploidization. In addition, use of a pharmacological inhibitor of IGF2BP3 expression elicited adult features in neonatal MKs. This could open the way to the development of new strategies to enhance MK maturation and platelet production. Other molecules favoring endomitosis have been identified, such as actin polymerization inhibitors and Rho kinase inhibitors (44–46), but these agents did not significantly improve platelet yields.
- DMS expansion. The production by a single MK of thousands of platelets requires considerable membrane synthesis and its folding into a well-organized DMS. The DMS is fueled by invagination from the outer membrane with further contributions from internal golgi-derived membranes and contacts with the endoplasmic reticulum, which together provide a continuous membrane supply (4). It has been reported that immature MKs have a high capacity for cholesterol and phospholipid synthesis and are also able to capture fatty acids (47). The importance of cholesterol uptake is further suggested by studies showing that hypercholesterolemia positively influences platelet production (48). A better knowledge of the lipid pathways involved in MK maturation could help us to devise culture media supplements favoring platelet production.

Bone marrow is a complex and dynamic cellular tissue where MKs interact with other cells and protein matrices in a 3-dimensional (3D) configuration (49). Recent findings have highlighted the environmental stiffness of the bone marrow as a key regulator of MK maturation, whereby adaptation of the cells to the surrounding physical constraints favors higher ploidy and proplatelet formation (34, 50). These observations can be applied directly to *in vitro* platelet production. Thus, Aguilar et al. recently showed that MKs grown in 2% methylcellulose (30–60 Pa) exhibited enhanced DMS expansion leading to increased platelet production. Mechanistically, these authors demonstrated the increased nuclear translocation of an important regulator of MK maturation, megakaryoblastic leukemia factor-1 (MKL1), which was triggered by the physical constraints (51). Identifying the stiffness-mediated factors involved in MK maturation should provide an important means of improving the production of platelets in culture.

## Efficiently releasing platelets from mature MKs

*In vivo*, under physiological conditions, efficient platelet release requires i) the transmigration of proplatelets/MK fragments through the endothelial barrier and ii) their exposure to the blood flow (8).

- Endothelial cells. Upon reaching the sinusoids, mature MKs come into contact with endothelial cells. A seminal study conducted by Rafii et al. demonstrated that human BM microvascular endothelial cells (BMECs) specifically supported the MK differentiation of CD34+ progenitors (52). More recently, the interplay between MKs and the sinusoidal barrier has been examined in more detail. It could be shown that MKs form podosomes which are able to degrade the extracellular matrix, allowing elongation of proplatelets into the lumen (53). The importance of podosomes in thrombopoiesis is further suggested by the occurrence of thrombocytopenia in primary genetic deficiencies affecting podosome formation (WASP, CDc42, α-actinin, or CD44) (54, 55). Work by Antkowiack et al. also indicates that EC contacts triggering podosome formation could participate in the DMS polarization preceding proplatelet extension (56).
  Current efforts in bioreactor development will require additional research to reveal the specific mechanisms involved in the transmigration of mature MKs into the lumen. Endothelial cells have already been introduced into 3D flow systems but did not appear to positively affect proplatelet elongation or platelet numbers (34, 57). The positive impact of EC might depend on the additional presence of soluble factors such as Il1b, an inflammatory cytokine reported to enhance MK and EC interactions (58). Stimulating the endothelium through the VEGFR1-mediated pathway also increased platelet production (59). Finally, a signaling lipid circulating in the blood, S1P, has been proposed as a new key regulator of platelet release, *in vitro* and under flow conditions (60).
- Blood flow. When left under static conditions, MKs extending proplatelets liberate individual platelets with a very low efficiency (61). Thus, to mimic *in vivo* conditions where platelet release depends heavily on shear forces (8, 60), a number of laboratories have integrated flow into newly developed scalable microfluidic platelet bioreactors. Baruch et al. have designed a bioreactor comprising a multitude of staggered pillars covered with von Willebrand factor. MKs adhering to the pillars and subjected to hydrodynamic forces stretch out long extensions and release platelet-like elements (62). Another microchamber developed by Thon et al. consists of one channel separated by a series of 2 μm diameter gaps from another channel where flow is applied. Trapped MKs extend proplatelets and release platelets into the second channel more efficiently than in the absence of flow (63). Nakagawa et al. have designed original gaps where trapped MKs are submitted to a bidirectional flow, applied at a 60° angle reported to be 3.6 times more effective than a 90° platelet release angle (64). Although this is an attractive variant, incorporation of this geometry into future devices will require thorough morphological and functional analysis of the platelets released. The development of these bioreactors has established proof of the feasibility of *ex vivo* platelet production. However, despite taking into account extracellular matrix proteins, stiffness, and flow, the platelet yields obtained are only of the order of 30–50 platelets/MK.

## Demonstrating native functionality and hemostatic properties following transfusion

If cultured platelets are to be considered as a clinical alternative, they must equal the quality of donor-derived platelets in terms of i) morphology and ultrastructure and ii) *in vitro*, and iii) *in vivo* functionality (65). So far, studies of the quality and functionality of cultured platelets are still fragmentary.

- Morphology and ultrastructure. Native platelets are typically anucleate, exhibit a characteristic discoid shape and are filled with secretory granules (α, δ, lysosomes) which contain endogenously synthesized or endocytosed molecules. Microscopic analyses have revealed that cultured platelets are typically larger than native ones and have an increased RNA content, two characteristic features of “young” platelets (27, 32, 35, 57). This raises the question of whether such immaturity is useful or detrimental for platelet recirculation after transfusion. In addition, platelet functions are largely dependent on the molecules stored in their granules. Culture media do not usually contain certain components required for platelet function such as fibrinogen or serotonin. Studies will be required to determine whether we need to load platelets with proteins they lack during culture, or whether they are capable of filling their granules during recirculation to ensure their normal function.
- *In vitro* evaluation of platelet function. In the majority of studies, the functionality of the platelets generated *in vitro* has only been incompletely addressed. The tests have mostly relied on flow cytometric measurement of P-selectin exposure and PAC-1 binding to detect GPIIb-IIIa activation. Usually, a large proportion of cultured platelets express these activation markers upon stimulation by agonists such as ADP or thrombin (27, 32, 34). One may note that a state of pre-activation, visualized by P-selectin expression, is often observed in the absence of any agonist (66, 67). In itself, this positivity does not inevitably predict poor transfusion properties. Indeed, in one study it was reported that circulating degranulated platelets rapidly lose surface P-selectin to the plasma pool but continue to circulate and function *in vivo* (68). The demonstration of platelet aggregation has often been restricted to a flow cytometric approach (2 color assays), due to the limited numbers of purified platelets obtained in culture (27, 34). However, as shown by Feng et al., this should not routinely exempt us from standard aggregometry testing as a quality control for transfusion applications (32).
- *In vivo* evaluation of platelet function. The *in vivo* functionality of cultured platelets has mainly been attested on the basis of their ability to participate in a developing thrombus after vessel injury in the mouse (27, 32, 35). Concerning their capacity to recirculate, this has only been demonstrated in a few studies. A quite similar half-life to that of native platelets was observed after transfusion into immune-deficient mice (27, 28, 32). Although these results are encouraging, they do not provide a definitive answer to the question of the true ability of cultured platelets to fulfill their functions. Finally, there are to date no available data concerning the *in vivo* hemostatic properties of the cells, i.e., their capacity to correct a bleeding tendency in thrombocytopenic individuals. With the declared ambition of being a transfusion substitute, it is now time to move on in the area of the functionality of cultured platelets with an accurate evaluation of their hemostatic potential in thrombocytopenic mice.

## Conclusion and perspectives

**Figure 2:**
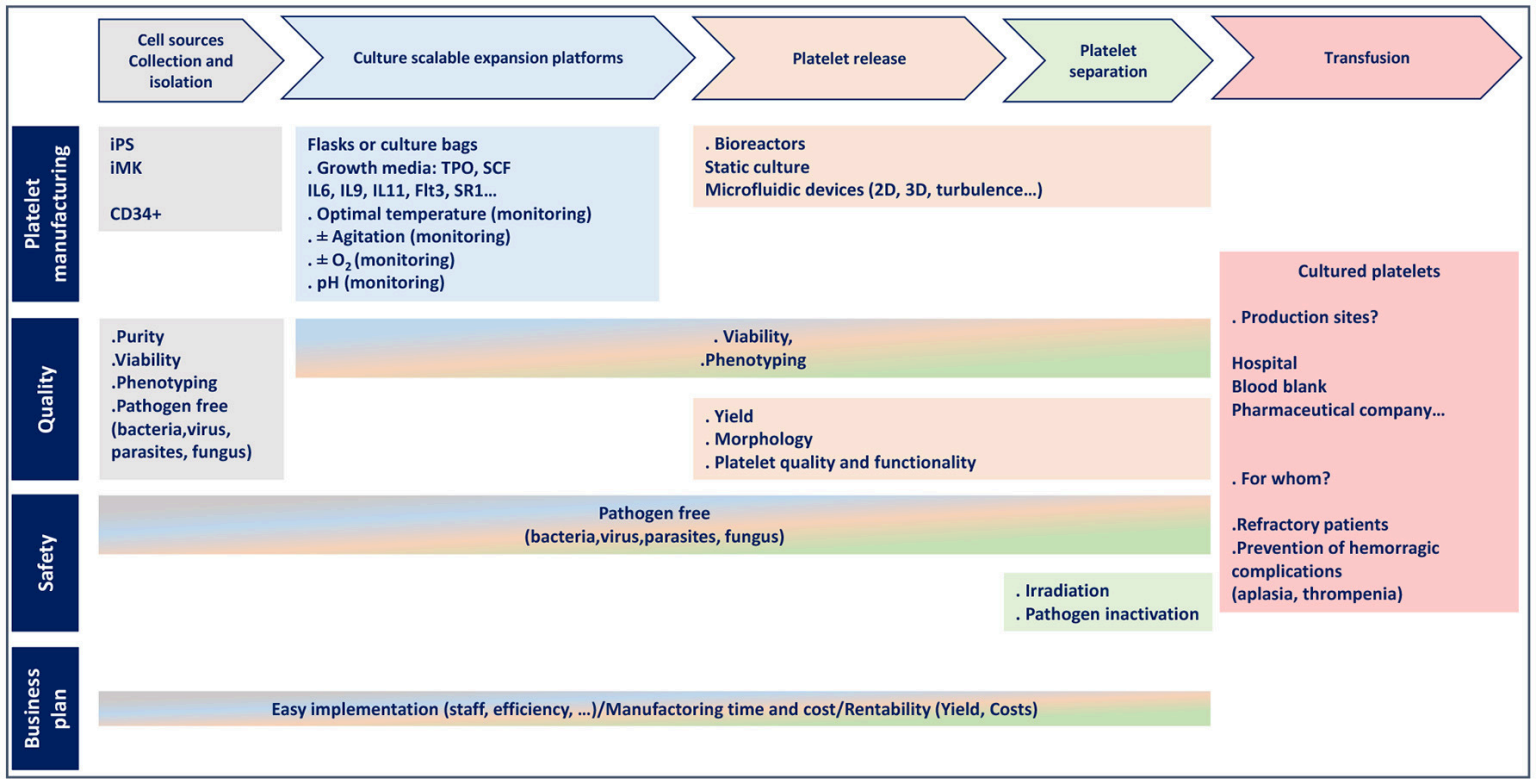
Flowchart for platelet manufacturing.

Over the past 5 years, considerable efforts have been made to improve the production of platelets *in vitro*, mainly in relation to iPSC generation and the availability of universal platelets, together with the design of original and scalable bioreactors. A dozen dedicated teams around the world are competing to achieve *ex vivo* platelet production. However, in addition to their important research efforts, it is also important to consider production costs which have to be greatly reduced to make cultured platelet an economic reality (Figure 2). In this respect, optimization of the crucial steps of platelet generation (MK maturation and platelet release) and a better understanding of the molecular and cellular mechanisms governing platelet production will be required to make cultured platelets a clinical alternative in certain situations. In any case, one must recall that despite the technical advances and enthusiasm underlying this challenge, nothing will ever replace the voluntary, free and anonymous donation of blood.

## Author contributions

All authors listed have made a substantial, direct and intellectual contribution to the work, and approved it for publication.

### Conflict of interest statement

The authors declare that the research was conducted in the absence of any commercial or financial relationships that could be construed as a potential conflict of interest.
